# Metabolic and inflammatory biomarkers are associated with epigenetic aging acceleration estimates in the GOLDN study

**DOI:** 10.1186/s13148-018-0481-4

**Published:** 2018-04-18

**Authors:** Marguerite R. Irvin, Stella Aslibekyan, Anh Do, Degui Zhi, Bertha Hidalgo, Steven A. Claas, Vinodh Srinivasasainagendra, Steve Horvath, Hemant K. Tiwari, Devin M. Absher, Donna K. Arnett

**Affiliations:** 1 Department of Epidemiology, University of Alabama at Birmingham, 1665 University Blvd, RPHB 230J, Birmingham, AL 35294 USA; 2 School of Biomedical Informatics, University of Texas Health Science Center at Houston, Houston, TX USA; 3 College of Public Health, University of Kentucky, Lexington, KY USA; 4 Department of Biostatistics, University of Alabama at Birmingham, Birmingham, AL USA; 5 Human Genetics, David Geffen School of Medicine, University of California Los Angeles, Los Angeles, CA 90095 USA; 6 Biostatistics, School of Public Health, University of California Los Angeles, Los Angeles, CA 90095 USA; 7 Human Genetics, Gonda Research Center, David Geffen School of Medicine, University of California Los Angeles, Los Angeles, CA 90095-7088 USA; 8 HudsonAlpha Institute for Biotechnology, Huntsville, AL USA

**Keywords:** Epigenetics, Epigenetic age, Age acceleration, Inflammation, Inflammatory markers, Lipids, Postprandial, Cholesterol, Triglycerides, Adiponectin

## Abstract

**Background:**

Recently, epigenetic age acceleration—or older epigenetic age in comparison to chronological age—has been robustly associated with mortality and various morbidities. However, accelerated epigenetic aging has not been widely investigated in relation to inflammatory or metabolic markers, including postprandial lipids.

**Methods:**

We estimated measures of epigenetic age acceleration in 830 Caucasian participants from the Genetics Of Lipid Lowering Drugs and diet Network (GOLDN) considering two epigenetic age calculations based on differing sets of 5′-Cytosine-phosphate-guanine-3′ genomic site, derived from the Horvath and Hannum DNA methylation age calculators, respectively. GOLDN participants underwent a standardized high-fat meal challenge after fasting for at least 8 h followed by timed blood draws, the last being 6 h postmeal. We used adjusted linear mixed models to examine the association of the epigenetic age acceleration estimate with fasting and postprandial (0- and 6-h time points) low-density lipoprotein (LDL), high-density lipoprotein (HDL), and triglyceride (TG) levels as well as five fasting inflammatory markers plus adiponectin.

**Results:**

Both DNA methylation age estimates were highly correlated with chronological age (*r* > 0.90). We found that the Horvath and Hannum measures of epigenetic age acceleration were moderately correlated (*r* = 0.50). The regression models revealed that the Horvath age acceleration measure exhibited marginal associations with increased postprandial HDL (*p* = 0.05), increased postprandial total cholesterol (*p* = 0.06), and decreased soluble interleukin 2 receptor subunit alpha (IL2sRα, *p* = 0.02). The Hannum measure of epigenetic age acceleration was inversely associated with fasting HDL (*p* = 0.02) and positively associated with postprandial TG (*p* = 0.02), interleukin-6 (IL6, *p* = 0.007), C-reactive protein (C-reactive protein, *p* = 0.0001), and tumor necrosis factor alpha (TNFα, *p* = 0.0001). Overall, the observed effect sizes were small and the association of the Hannum residual with inflammatory markers was attenuated by adjustment for estimated T cell type percentages.

**Conclusions:**

Our study demonstrates that epigenetic age acceleration in blood relates to inflammatory biomarkers and certain lipid classes in Caucasian individuals of the GOLDN study. Future studies should consider epigenetic age acceleration in other tissues and extend the analysis to other ethnic groups.

## Background

Increasing age is a major risk factor for chronic disease. Understanding risk factors for accelerated aging can help target strategies aimed at preventing or delaying onset of age-related disease [1, 2]. Importantly, the epigenome is an intriguing target for age-related physiological changes, as it is modifiable by the environment and is a major determinant of gene expression [3]. Recently, calculated “epigenetic age” has become a novel aging biomarker, which has been demonstrated to accurately predict chronological age across a broad spectrum of tissues and cell types [4]. Epigenetic age acceleration—or older epigenetic age in comparison to chronological age—has been robustly associated with all-cause mortality [5], obesity [6], physical fitness [7], and stress [8]. However, accelerated epigenetic aging has not been evaluated in relation to intermediate phenotypes such as postprandial blood lipid levels, inflammatory markers, or adiponectin.

Of those, postprandial blood lipid levels are especially relevant given that most people spend the majority of the day in a postprandial state [9]. Results from basic and clinical studies suggest elevated and prolonged postprandial lipemia is associated with prothrombotic and proinflammatory processes that may precipitate cardiovascular disease (CVD) and aging [10, 11]. In the current study, we evaluated the association of two measures of epigenetic age acceleration [4, 12] with fasting and postprandial lipids, five inflammatory markers, and adiponectin, using data from the Genetics of Lipid Lowering Drugs and Diet Network (GOLDN) study.

## Methods

### Study population

The GOLDN study determined genetic predictors of participant lipid and inflammatory response to diet and fenofibrate treatment interventions [13–15]. Participants were identified through 3-generation families previously screened in the National Heart, Lung and Blood Institute Family Heart Study (FHS) Minnesota and Utah centers. Individuals who used lipid-lowering drugs at the time of screening were required to discontinue them for 4 weeks prior to study participation. Participants fasted for at least 8 h and abstained from alcohol intake for ≥ 24 h. The postprandial lipemia intervention followed the protocol of Patsch et al. [16]. The whipping cream (83% fat) meal had 700 calories/m^2^ body surface area; 3% of calories were derived from protein, and 14% from carbohydrate. Blood samples were drawn immediately before the meal with the last draw being 6 h later. During the 6-h study period, participants consumed only water and abstained from physical activity. Inflammatory markers and adiponectin were assayed only in the fasting state. The data used in this analysis were collected at the time of the postprandial lipemia challenge and, thus, except for the 6-h lipid measures, was cross-sectional.

### Lipids, inflammatory markers, and adiponectin

Triglycerides (TG), high-density lipoprotein (HDL), low-density lipoprotein (LDL), and total cholesterol were measured by nuclear magnetic resonance (NMR) spectroscopy at fasting and during the postprandial intervention (Liposcience, Raleigh, NC) [17]. High-sensitivity C-reactive protein (hsCRP) was measured on the Hitachi 911 using a latex particle-enhanced immunoturbidimetric assay (Kamiya Biomedical Company, Seattle, WA). Interleukin-6 (IL6), interleukin-2 soluble receptor alpha (IL2sRα), tumor necrosis factor (TNF)-α and monocyte chemoattractant protein-1 (MCP1) were measured using quantitative sandwich enzyme immunoassays (ELISA kit assays, R&D Systems Inc., Minneapolis, MN) as described in previous publications [18, 19]. Adiponectin was measured using an ELISA kit from R&D Systems (Minneapolis, MN) [20].

### DNA methylation measurements and epigenetic age calculation

DNA from a total 993 GOLDN participants was assayed using Illumina’s Infinium HumanMethylation450 Beadchip as previously described by Irvin et al. [21]. Briefly, CD4+ T cells were harvested from stored buffy coats (from the fasting sample) using antibody-linked Invitrogen Dynabeads [22]. Cells were lysed and DNA was extracted using DNeasy kits (Qiagen, Venlo, Netherlands). After following standard Illumina protocols, the resulting intensity files were analyzed with Illumina’s GenomeStudio to generate beta scores representing the percent of methylated sites at that location across all the cells in a sample. After previously reported QC, methylation data were available for 461,281 5′-cytosine-phosphate-guanine-3′ genomic site (CpGs) [21]. The non-normalized data were uploaded into the DNA methylation age calculator [4] using the advanced analysis in blood option. We removed 140 samples for which the predicted tissue was not blood. To err on the side of caution, we removed 23 individuals from the analysis because they were severe outliers according to the Horvath measure of epigenetic age acceleration (> 4 standard deviations away from the mean), leaving a total sample size of *n* = 830 individuals for the analysis.

**Fig. 1:**
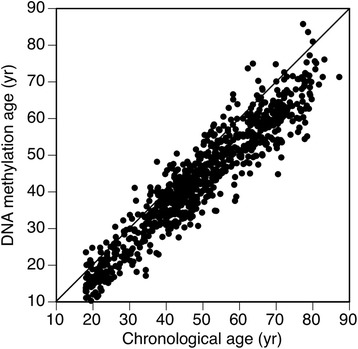
Calculated (Horvath method) epigenetic age versus chronological age in GOLDN

**Fig. 2:**
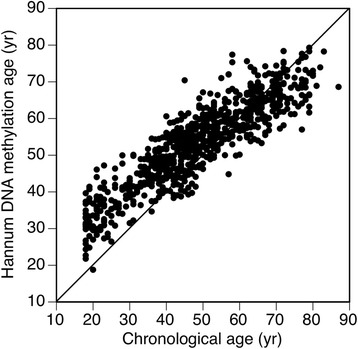
Calculated (Hannum method) epigenetic age versus chronological age in GOLDN

In addition to the Horvath measure of epigenetic age acceleration, we considered another independent measure of age acceleration derived from the Hannum methylation age estimate [12]. Both methylation age acceleration estimates have been associated with outcomes in a side-by-side manner in previous publications [5, 23, 24]. The Horvath methylation age estimate (DNAm age) is based on 353 CpGs and has been robustly correlated with chronological age in multiple studies. DNAm age sometimes systematically underestimates chronological age [25]. To remove this systematic offset from the analysis, this study leverages epigenetic age acceleration defined by regressing DNAm age on chronological age and forming residuals. The second estimate is based on Hannum’s methylation age estimate calculated from 71 CpGs. Figures 1 and 2 are the scatter plots of calculated epigenetic age versus chronological age for the Horvath and Hannum calculators, respectively, in GOLDN. The Horvath and the Hannum measures of DNAm age are related, but they capture slightly different aspects of biology (only 6 CpGs overlap) [5]. The Hannum age estimator is correlated with estimated blood cell counts, which reflects that it was constructed on the basis of whole blood DNA methylation data [5, 26]. In the current analysis, we focused on a modification of the Hannum estimate described (in previous publications) as *extrinsic* epigenetic age acceleration (EEAA) since it has been found to outperform the original Hannum measure when it comes to predicting lifespan [5]. EEAA is a residual formed from regressing the Hannum estimate weighted by the proportion of naive CD8+ T cells, exhausted CD8+ T cells, and plasmablasts on chronological age which makes the estimate more strongly reflective of immune system aging [5, 23, 27]. Chen et al. reported the Hannum estimator tracks aspects of immunosenescence exhibiting significant (albeit weak) correlations with several markers of immunosenescence, e.g., the abundance of senescent (or conversely naïve) T cells and telomere length [26, 27]. The Horvath DNAm age estimator exhibits substantially weaker correlations with estimated blood cell counts, which reflects the fact that it was constructed across a broad spectrum of tissues and cell types. Overall, the Horvath DNAm age estimator does not relate to measures of immunosenescence (including telomere length) [27]. Rather, it mostly captures cell intrinsic DNAm age changes which might reflect the actions of an innate aging process.

For both methylation age acceleration estimates, a positive value indicates that the epigenetic age is higher than expected based on chronological age (i.e., accelerated epigenetic aging). We note EEAA in GOLDN is limited by the use of DNA methylation data derived from CD4+ T cells as opposed to peripheral blood mononuclear cells (PBMCs) like in previous studies. For a more direct comparison to the Horvath estimate we also report the results for the Hannum residual defined by regressing the (unweighted) Hannum methylation age estimate on chronological age in a secondary analysis.

### Statistical analysis

Participant characteristics were compared by the median of each age acceleration measure using chi square tests and t-tests, as appropriate. TG, hsCRP, IL6, IL2sRα, TNFα, MCP1, and adiponectin were ln-transformed to achieve normality. Pearson correlation coefficients between lipids, inflammatory markers, adiponectin, and each measure of age acceleration were also estimated. We used linear mixed models to examine the association of the Horvath epigenetic age acceleration residual with fasting and postprandial total cholesterol, LDL, HDL, and TG as well as inflammatory markers and adiponectin (as outcomes). The model was adjusted for age, study site, sex, corresponding fasting lipid level (if applicable), deconvolution estimated T cell type (naïve, regulatory, memory) percentages (estimated using a method developed by the GOLDN group [28]), age acceleration estimate squared (to account for a potential non-linear effect of this variable), current smoking status, current drinking status and a random effect of family relationship. Linear mixed models were implemented in the R *kinship* package (*lmekin* function) [29]. For our analysis of the EEAA estimate we used a similar model, except the model was not adjusted for estimated T cell type percentages since blood cell composition is a component of the age acceleration estimate. For both the Horvath and Hannum estimates we considered a sensitivity model adjusted for history of coronary heart disease (CHD) and diabetes status. Further, we explored a gender by age acceleration interaction term. In total we tested the association of 14 outcomes with each age acceleration estimate making the Bonferroni correction for multiple testing *p* = 0.003 (0.05/14). In secondary analysis we examined the association of each outcome with the Hannum residual, considering the model described above for EEAA both with and without adjustment for T cell percentages.

## Results

The mean and standard deviation of the Horvath and Hannum (EEAA) estimates were − 3.54 (6.1) and − 1.32 (8.3), respectively. The Pearson correlation coefficient between methylation and chronological age was 0.94 (*p* < 0.0001) for the Horvath estimate and 0.91 (*p* < 0.0001) for the Hannum (EEAA) estimate. The correlation coefficient between the Horvath and Hannum (EEAA) age acceleration measures was 0.50 (*p* < 0.0001). Table 1 shows descriptive statistics of the GOLDN population, categorized by the median of each age acceleration value (− 3.33 and − 1.64 for the Horvath and Hannum (EEAA) estimates, respectively). There was a higher prevalence of current drinking, history of CVD, hypertension, and diabetes associated with being above the median for the Horvath age acceleration measure. Similarly, being above the median of the age acceleration estimate for the Hannum method was associated with history of CVD, hypertension, and diabetes, but not with current drinking. Increasing age acceleration as measured by the Horvath method was associated with increased LDL, TC, and TG in the fasting and postprandial states. Having an age acceleration estimate above the median for the Hannum method was associated with decreased HDL and increased TG in the fasting and postprandial states. The Hannum measure of age acceleration (but not the Horvath measure) was also associated with increased C-reactive protein (CRP), IL2sRα, tumor necrosis factor alpha (TNFα), and MCP1 (Table 1). Table 2 shows the unadjusted correlation coefficients for each age acceleration estimate with lipid classes, inflammatory markers, and adiponectin. Each age acceleration measure was associated with lipid classes in the fasting and postprandial state except the Horvath measure was not associated with HDL. Each measure of age acceleration was inversely associated with adiponectin. The Hannum age acceleration estimate but not the Horvath estimate was positively associated with more than one inflammatory marker.

**Table 1:** Descriptive statistics of the GOLDN study population categorized by the median of two age acceleration residuals

	Horvath age acceleration			Hannum age acceleration (EEAA)		
*N* (%) or mean (SD)	Below median	Above median	*p*	Below median	Above median	*p*
Center (UT)	220 (53.0)	153 (36.9)	< 0.0001	223 (46.2)	192 (43.6)	0.44
Female	226 (54.5)	193 (46.5)	0.02	222 (53.5)	197 (47.5)	0.08
Current smoker	34 (8.2)	35(8.4)	0.89	32 (7.7)	37 (8.9)	0.52
Current drinking	201 (48.4)	238 (57.4)	0.01	228(54.9)	211 (50.8)	0.23
History of CHD	16 (3.9)	27 (6.5)	0.08	15 (3.6)	28 (6.8)	0.04
Hypertension	99 (23.9)	121 (29.2)	0.08	89 (21.5)	131 (31.6)	0.001
Diabetes	22 (5.3)	41 (9.8)	0.01	23 (5.5)	40 (9.6)	0.03
Age	46.6 (16.2)	50.7 (15.5)	0.0002	46.6 (16.1)	50.7 (15.6)	0.0002
Lipids						
LDL mg/dL (0 h)	114.9 (31.8)	121.7 (32.2)	0.003	116.5 (31.8)	120.1 (32.4)	0.10
LDL mg/dL (6 h)	119.2(32.1)	125.9 (33.2)	0.003	120.8 (32.2)	124.4 (33.3)	0.12
HDL mg/dL (0 h)	46.0 (14.1)	44.5 (13.1)	0.12	47.1 (14.5)	43.4 (12.4)	< 0.0001
HDL mg/dL (6 h)	48.8 (14.6)	47.8 (14.1)	0.34	50.2 (15.2)	46.3 (133)	< 0.0001
TC mg/dL (0 h)	181.6 (38.4)	189.7 (40.0)	0.003	183.9 (38.8)	187.4(39.9)	0.20
TC mg/dL (6 h)	189.2 (39.6)	199.0 (42.9)	0.0007	191.9 (40.3)	196.3 (42.7)	0.13
TG mg/dL (0 h)	135.5 (84.8)	155.5 (108.5)	0.003	133.4 (83.0)	157.8 (109.5)	0.0003
TG mg/dL (6 h)	187.9 (130.5)	230.8 (166.8)	< 0.0001	187.0 (129.7)	231.8 (167.3)	< 0.0001
Inflammatory markers						
IL6	1.9 (2.2)	2.2 (4.4)	0.11	1.8 (26)	2.2 (4.2)	0.08
CRP	0.21 (0.3)	0.25 (0.4)	0.22	0.20 (0.4)	0.26 (0.4)	0.03
MCP1	207.8 (62.5)	211.2 (68.7)	0.45	204.3 (64.9)	214.7 (66.1)	0.02
IL2SRA	1012.3 (344.5)	1021.2 (377.1)	0.72	991.2 (330.3)	1042.3 (388.0)	0.04
Adiponectin	8498.6 (4644.7)	8047.9 (4827.6)	0.17	8568.1 (4746.8)	7978.2 (4719.4)	0.07
TNFα	3.2 (1.9)	3.9 (8.0)	0.08	3.1 (1.8)	4.0 (8.0)	0.02

*EEAA extrinsic* epigenetic age acceleration, *CHD* coronary heart disease, *CRP* C-reactive protein, *DNAmage* DNA methylation age, *HDL* high-density lipoprotein, *IL2SRA* interleukin-2 soluble receptor alpha, *IL6* interleukin 6, *LDL* low-density lipoprotein, *MCP1* monocyte chemotactic protein 1, *TC* total cholesterol, *TG* triglyceride, *TNFa* tumor necrosis factor alpha

**Table 2:** Marginal correlations between lipid and other biomarkers with epigenetic age acceleration in the GOLDN study

	Horvath age acceleration		Hannum age acceleration (EEAA)	
	*r*	*p*	*r*	*p*
Lipids				
LDL mg/dL (0 h)	0.11	0.0008	0.11	0.002
LDL mg/dL (6 h)	0.12	0.0009	0.10	0.003
HDL mg/dL (0 h)	− 0.02	0.50	− 0.13	0.0002
HDL mg/dL (6 h)	− 0.01	0.83	− 0.12	0.0004
TC mg/dL (0 h)	0.12	0.0004	0.08	0.02
TC mg/dL (6 h)	0.14	< .0001	0.09	0.007
TG mg/dL (0 h)	0.11	0.001	0.11	0.002
TG mg/dL (6 h)	0.17	< .0001	0.15	< .0001
Inflammatory markers				
IL6 (0 h)	0.05	0.18	0.09	0.02
CRP (0 h)	0.04	0.25	0.06	0.09
MCP1 (0 h)	0.01	0.81	0.07	0.05
IL2SRA (0 h)	−0.01	0.84	0.08	0.01
Adiponectin (0 h)	− 0.08	0.03	− 0.07	0.05
TNFα (0 h)	0.05	0.16	0.11	0.002

*EEAA extrinsic* epigenetic age acceleration, *0 h* fasting time point, *6 h* postprandial time point

Regression results for the Horvath and Hannum age acceleration estimates are presented in Table 3. The Horvath epigenetic age acceleration residual was not associated with fasting lipid levels. Increasing age acceleration residual was marginally associated with higher postprandial HDL (*p* = 0.05) and postprandial total cholesterol (*p* = 0.06) after adjustment. The effect size for these associations is 0.30 mg/dL and 0.92 mg/dL for postprandial HDL and TC respectively for each 1 standard deviation change in the age acceleration estimate. The Horvath age acceleration estimate was also inversely associated (albeit marginally) with IL2sRα. The Hannum EEAA measure was inversely associated with fasting (*p* = 0.02) but not postprandial HDL levels, and was positively associated with postprandial (*p* = 0.02) but not fasting TG. The Hannum measure of epigenetic age acceleration was also strongly positively associated with IL6 (*p* = 0.007), CRP (*p* = 0.0001), and TNFα (*p* = 0.0001). The association of EEAA with CRP and TNFα were the only two associations that reached statistical significance after correction for multiple testing in Table 3. The ratio of postprandial TG, IL6, CRP, and TNFα associated with each 1 standard deviation increase in EEAA was 1.03, 1.06, 1.16, and 1.06, respectively. Additionally, adjusting for history of CHD and diabetes status in these models did not substantially change our results (data not shown). We did not find evidence of effect modification by gender of the relationship between epigenetic age acceleration and our traits except for CRP, where CRP was associated with epigenetic age acceleration (EEAA estimate) only in females (*p* = 0.048). This result does not pass significance criteria after correction for multiple testing.

**Table 3:** Multivariable linear regression models that relate biomarkers (rows) to epigenetic age acceleration (covariate) using the Horvath calculator and the Hannum calculator

	Horvath age acceleration			Hannum age acceleration (EEAA)		
Dependent variable	Beta*	SE	*p*	Beta#	SE	*p*
LDL mg/dL (0 h)	0.36	0.21	0.09	0.166	0.12	0.17
LDL mg/dL (6 h)	0.003	0.06	0.96	− 0.006	0.04	0.87
HDL mg/dL (0 h)	0.06	0.08	0.52	− 0.11	0.04	*0.02*
HDL mg/dL (6 h)	0.05	0.03	*0.05*	0.004	0.15	0.81
TC mg/dL (0 h)	0.44	0.25	0.08	0.091	0.14	0.52
TC mg/dL (6 h)	0.15	0.08	0.06	0.068	0.05	0.13
TG mg/dL (0 h)	0.001	0.004	0.75	0.002	0.002	0.30
TG mg/dL (6 h)	0.0005	0.002	0.84	0.004	0.001	*0.02*
IL6 (0 h)	− 0.001	0.004	0.76	0.007	0.002	*0.007*
CRP (0 h)	0.007	0.008	0.34	0.018	0.004	*0.0001*
MCP1 (0 h)	− 0.001	0.002	0.55	− 0.0003	0.001	0.76
IL2SRA(0 h)	− 0.006	0.002	*0.02*	0.002	0.001	0.09
Adiponectin (0 h)	− 0.003	0.004	0.46	− 0.003	0.002	0.24
TNFα (0 h)	− 0.0008	0.003	0.80	0.007	0.002	*0.0001*

Since the models include a quadratic term the effect of a one unit change in *x* on *y* is not constant

*EEAA extrinsic* epigenetic age acceleration, *0 h* fasting time point, *6 h* postprandial time point, *CRP* C-reactive protein, *HDL* high-density lipoprotein, *IL2SRA* interleukin-2 soluble receptor alpha, *IL6* interleukin-6, *LDL* low-density lipoprotein, *MCP1* monocyte chemotactic protein 1, *SE* standard error, *TC* total cholesterol, *TG* triglyceride, *TNFa* tumor necrosis factor alpha

*Model is adjusted for adjusted for age, study site, sex, fasting lipid level (if applicable), estimated T cell type percentages, epigenetic age acceleration estimate squared (i.e., a quadratic polynomial), current smoking status, current drinking status, and a random effect of family relationship

#Model is adjusted for adjusted for age, study site, sex, fasting lipid level (if applicable), epigenetic age acceleration estimate squared (i.e., a quadratic polynomial), current smoking status, current drinking status, and a random effect of family relationship

In secondary analysis of the Hannum residual (Table 4), the results were consistent with those presented for EEAA in Table 3 except that the Hannum residual was not associated with IL6. After T cell type percentages were included in the model, the association with fasting HDL, CRP, and TNFα was attenuated. Postprandial TG remained marginally (0.08) associated with the Hannum residual even after adjustment for T cell type percentages.

**Table 4:** Multivariable linear regression models that relate biomarkers (rows) to epigenetic age acceleration (covariate) using the Hannum residual with and without adjustment for estimated T cell type percentages

	Hannum residual					
	Model 1			Model 2		
Dependent variable	Beta	SE	*p*	Beta	SE	*p*
LDL mg/dL (0 h)	0.12	0.14	0.39	0.12	0.15	0.41
LDL mg/dL (6 h)	0.003	0.04	0.94	0.02	0.05	0.71
HDL mg/dL (0 h)	− 0.12	0.05	*0.03*	− 0.06	0.06	0.32
HDL mg/dL (6 h)	0.002	0.02	0.91	0.0008	0.02	0.97
TC mg/dL (0 h)	0.08	0.16	0.60	0.10	0.17	0.56
TC mg/dL (6 h)	0.07	0.05	0.16	0.07	0.05	0.17
TG mg/dL (0 h)	0.004	0.002	0.10	0.003	0.003	0.23
TG mg/dL (6 h)	0.003	0.002	*0.03*	0.003	0.002	0.08
IL6 (0 h)	0.002	0.003	0.53	− 0.002	0.003	0.46
CRP (0 h)	0.01	0.005	*0.005*	0.007	0.006	0.20
MCP1 (0 h)	− 0.00008	0.001	0.95	0.0006	0.001	0.62
IL2SRA (0 h)	− 0.00006	0.002	0.97	− 0.0004	0.002	0.79
Adiponectin (0 h)	− 0.003	0.002	0.20	− 0.0010	0.003	0.72
TNFα (0 h)	0.004	0.002	*0.04*	0.002	0.002	0.37

Model 1 is adjusted for adjusted for age, study site, sex, fasting lipid level (if applicable), epigenetic age acceleration estimate squared (i.e., a quadratic polynomial), current smoking status, current drinking status, and a random effect of family relationship. Model 2 adds estimated T cell type percentages to model 1. Since the models include a quadratic term, the effect of a one unit change in *x* on *y* is not constant

*0 h* fasting time point, *6 h* postprandial time point, *CRP* C-reactive protein, *HDL* high-density lipoprotein, *IL2SRA* interleukin-2 soluble receptor alpha, *IL6* interleukin 6, *LDL* low-density lipoprotein, *MCP1* monocyte chemotactic protein 1, *SE* standard error, *TC* total cholesterol, *TG* triglyceride, *TNFa* tumor necrosis factor alpha

## Discussion

Measures of epigenetic age acceleration are robust biomarkers of aging independent of chronological age [4, 12]. In the current study, we tested the association of two different measures of epigenetic age acceleration with metabolic and inflammatory markers in 830 Genetics Of Lipid Lowering Drugs and Diet Network study participants. Similar to previous publications, epigenetic age acceleration was marginally associated with lipids, including postprandial lipids. We are the first to report that a measure of epigenetic age acceleration derived from the Hannum methylation age estimate (referred to as *extrinsic* epigenetic age acceleration) was strongly related to more than one inflammatory marker. However, further analysis showed this relationship was not independent of T cell type composition. Previous studies have reported each epigenetic age acceleration measure to be associated with outcomes including cancer mortality, cardiovascular disease mortality, and all-cause mortality [5, 30]. Continued research is needed to better understand if increased inflammation could be a pathway through which epigenetic aging is related to these outcomes.

A recent large meta-analysis of over 13,000 participants (including non-Hispanic whites, Hispanics, and African Americans) demonstrated that, independent of chronological age and traditional risk factors, epigenetic age acceleration assessed in blood (measured by both the Horvath and Hannum methods) predicted all-cause mortality [5]. Another study of ~ 2000 older Caucasian individuals from the ESTHER cohort (a large population-based epidemiologic study of chronic disease in Germany) reported that, for each 5 year increase of DNA methylation age (DNAmage, Horvath estimate) minus chronological age, the hazard ratio for all-cause mortality was 1.23 (95% CI 1.10–1.38), cancer mortality was 1.22 (95% CI 1.03–1.45), and cardiovascular mortality was 1.19 (95% CI 0.98–1.43) after adjustment for confounding factors [30]. Among ~ 1500 women from the Women’s Health Initiative (WHI) study in a report from Horvath et al., neither measure of epigenetic age acceleration was associated with coronary heart disease (CHD) [24]. In that study, the measure of epigenetic age acceleration derived from the Horvath calculator was not robustly associated with triglyceride levels, HDL, glucose, CRP, or body mass index (BMI, *p* > 0.05) after adjustment. The extrinsic epigenetic age acceleration estimate (Hannum) was associated with fasting TG (*p* = 0.04) and CRP (*p* = 0.02), but not fasting HDL (*p* = 0.80). In our study, the direction of association of the Horvath estimate with fasting HDL was positive (*p* = 0.52), while the Hannum EEAA estimate was negatively associated with fasting HDL (*p* = 0.02). In the report from Horvath et al. mentioned above, the direction of association for the Horvath and Hannum estimates with HDL also conflicted. Another study reported that older epigenetic age in comparison to chronological age was associated with increased BMI in middle-aged but not in older adults, suggesting that the relationship between age acceleration and metabolic traits may change over the lifespan [6].

Since elevated postprandial lipids are predictive of aging and inflammation [9, 10], we investigated whether epigenetic age acceleration was associated with postprandial lipids. Like fasting lipids and other cardiometabolic risk factors investigated in other studies, our results demonstrate that epigenetic age acceleration is a modest predictor of postprandial lipemia. None of the results for lipids presented in Table 3 (fasting or postprandial) were significant after correction for multiple testing. Notably, after adjustment EEAA was associated with higher levels of postprandial TGs (*p* = 0.02), which is the lipid class that changes the most upon fat loading. The Horvath measure of epigenetic age acceleration was marginally associated with higher postprandial TC. With PPL and methylation measured on a single day in GOLDN, it is difficult to determine if increased metabolic parameters could lead to accelerated epigenetic age or vice versa. Longitudinal PPL and methylation data would be necessary to untangle the temporal relationship between epigenetic age acceleration and postprandial lipids. Few other studies have investigated whether diet is linked to epigenetic age acceleration. One study including > 4000 postmenopausal female participants from the WHI, as well as 402 male and female participants from the Italian cohort study, Invecchiare nel Chianti, suggested poultry intake was protective for epigenetic aging. In that same study, fish intake, alcohol, and smoking were not associated with the epigenetic age acceleration residual as measured by the Horvath age calculator.

In GOLDN, the epigenetic age acceleration measure from Horvath’s calculator was associated with IL2sRα but not other inflammatory markers or adiponectin. On the other hand, EEAA was positively (but modestly) associated with three biomarkers of inflammation (IL6, CRP, and TNFα). Elevation of these three markers has been associated with a number of age-related chronic diseases [31]. Additionally, we considered the Hannum residual in a secondary analysis to help understand if the association between EEAA and inflammatory markers observed in our primary analysis could be due to a difference in probes across the two age acceleration measures or a function of the contribution of blood cell composition to the Hannum EEAA estimate. After adjustment for T cell type composition in a model considering the Hannum residual as the variable of interest, the three inflammatory markers were no longer associated with the Hannum estimate. In a previous study, EEAA was shown to be correlated with the sample estimated naïve and memory CD8^+^ T cell populations [27]. In that same study, adjustment for T cell composition in a model correlating EEAA to leukocyte telomere length attenuated the association. In our study, the Hannum residual and Hannum EEAA estimate were strongly correlated with estimated populations of naïve CD4+ T cell percentages (*r* = − 0.45, *p* < 0.0001 and − 0.62, *p* < 0.0001, respectively). Overall, this lends further support to the conclusions from Chen et al. which suggested that EEAA captures information on key aspects of immunosenescence, which may help explain the stronger relationship of EEAA with mortality observed in a previous study in comparison to the Horvath estimate [5].

The current study is set in one of few observational epidemiology cohort studies with epigenetic array data and a postprandial lipemia intervention on a sizable population. Participants in the GOLDN study are, on average, middle aged, and because the study required discontinuation of lipid-lowering therapy, the study population is relatively healthy. Studies of the relationship between postprandial lipemia and epigenetic aging are still warranted in other groups (e.g., older individuals, other ethnic groups) and in other tissues.

## Conclusion

Inflammatory markers associated with aging exhibit significant associations with a measure of epigenetic age acceleration. However, this association was not independent of other key aspects of immune system aging (estimated naïve T cell type percentages) in our study. The same measure of epigenetic age acceleration was weakly associated with increased postprandial triglyceride levels. Future studies should revisit these analyses by employing even larger sample sizes, different ethnic groups, and DNA methylation data from other tissues, such as saliva, which may be easier to collect.

## Abbreviations

BMI: Body mass index
CHD: Coronary heart disease
CpG: 5′-Cytosine-phosphate-guanine-3′ genomic site
CRP: C-reactive protein
CVD: Cardiovascular disease
DNAm age: DNA methylation age
GOLDN: Genetics of Lipid Lowering Drugs and Diet Network (study)
HDL: High-density lipoprotein
hsCRP: High-sensitivity C-reactive protein
IL2sRα: Interleukin-2 soluble receptor alpha
IL6: Interleukin-6
LDL: Low-density lipoprotein
MCP1: Monocyte chemoattractant protein-1
NMR: Nuclear magnetic resonance
PBMC: Peripheral blood mononuclear cell
TG: Triglycerides
TNFα: Tumor necrosis factor alpha
WHI: Women’s Health Initiative (study)
